# Affect and conation in second language learning: Survey data from Ukrainian learners of English

**DOI:** 10.1016/j.dib.2024.110090

**Published:** 2024-01-24

**Authors:** Gavin Austin, Andriy Yanovych

**Affiliations:** University of Southern Queensland, Ipswich campus, 11 Salisbury Rd, Ipswich, QLD, 4305, Australia

**Keywords:** Second language acquisition, Peer support, Foreign language anxiety, Willingness to communicate, Individual differences

## Abstract

This article presents a dataset concerned with second language (L2) learning [1]. We investigated two affective variables (i.e., peer support [PS] and foreign language anxiety [FLA]) and one conative variable (i.e., willingness to communicate [WTC]). A total of 387 adult Ukrainian learners of English (ULEs) completed an online survey. The main items in the survey were 22 Likert-type items, each with response options ranging from 1 = ‘strongly disagree’ to 5 = ‘strongly agree’. The dataset includes the numerical responses of the participants to these items, plus their genders and ages. It also includes the participants’ written responses to one open question. This dataset can be used to explore the correlational or causal relationships among PS, FLA and WTC in L2 learning.

Specifications Table

**Table:** 

Subject	Social science
Specific subject area	Affective and conative variables in L2 learning
Data format	Raw
Type of data	Table (.xlsx)
Data collection	The data were collected using an online survey consisting of 22 Likert-type items plus one open question. To measure PS, we used the five items in the Students Personal Support Scale in [2]. For FLA, the Foreign Language Classroom Anxiety Scale in [3] was used. This scale consists of eight items, two of which are reverse-coded, and is an abbreviated version of the 33-item scale created by Horwitz et al. [4]. WTC was measured using the nine-item scale in [5]. For each Likert-type item, the respondent was asked to indicate the extent to which they disagreed or agreed with a particular statement (e.g., “I am willing to ask the teacher a question in English in the classroom”). The response options were: 1 = ‘strongly disagree’, 2 = ‘disagree’, 3 = ‘neither disagree nor agree’, 4 = ‘agree’, 5 = ‘strongly agree’. A total of 387 ULEs took part: these were adult Ukrainian nationals who were either studying English at the time in a classroom setting, or had studied it in the past in this learning context. The participants were recruited via Facebook.
Data source location	Institution: University of Southern Queensland City/Town/Region: Ipswich Country: Australia Latitude and longitude (and GPS coordinates) for collected samples/data: 27.6146° S, 152.7608° E
Data accessibility	Repository name: OSF DOI:10.17605/OSF.IO/EJFTX
	Direct URL to dataset: https://osf.io/ejftx/

## 1 Value of the Data

• Although the role of peer support in L2 learning has received some attention from researchers, we know of no work that has been done in this area on ULEs specifically. Hence, a future study which uses the present dataset will help to extend our understanding of the role of peer support in L2 learning in cross-linguistic terms.
• The dataset can be used to explore the correlational or causal relationships among PS, FLA and WTC in L2 learning.
• The dataset may be useful to researchers who are interested in the role played by affect or conation in L2 learning.
• The dataset plus the associated files can be used as exemplars for a similar study in the future.
• The results of a future study based on the dataset may have implications for L2 classroom practice.

## 2 Data Description

The files associated with this dataset are as follows.

*1_data.xlsx* This file contains the raw data set. It includes 1714 responses to the survey (483 complete, 1231 incomplete). Of the 483 complete cases, 387 were used in the data analysis; the reason is given below. In addition to the participants’ responses to 22 Likert-type items, the file contains the following information: each participant's gender and age, the times when they started and finished the survey, their email address, and their response to one open question. Most of the responses to this question are in Ukrainian: only a handful are in English.
*2_preparation.Rmd* This file contains code in R [6]. The code was used to prepare the raw data set for analysis.
*3_outliers.Rmd* The R code in this file was used to identify outliers on the basis of Mahalanobis distance.
*4_assumptions.Rmd* The R code in this file was used to check the statistical assumptions that would be relevant to this data set if it were used for regression analysis (i.e., additivity, linearity, multivariate normality, homogeneity and homoscedasticity). Note that, for the additity assumption, we treat all three variables in the data set as potential predictor variables.
*5_reliability.Rmd* The R code in this file was used to check the reliability (i.e., internal consistency) of the survey items in each scale. We employed two measures of reliability: coefficient *α* and McDonald's *ω_._*
*6_summary.Rmd* The R code in this file was used to generate the means and standard deviations reported in Table 1, Table 2, Table 3 below, and to sort the items within each table.

**Table 1:** Peer support.

Item ID	Item	*M*	*SD*
PS3	Other students care about my feelings.	3.15	1.13
PS5	Other students really care about me.	2.96	1.10
PS1	Other students think it is important to be my friend.	2.84	1.08
PS4	Other students like me as much as they like others.	2.27	1.00
PS2	Other students like me the way I am.	2.17	1.05

**Table 2:** Foreign language anxiety.

Item ID	Item	*M*	*SD*
FLA8	I feel uncomfortable when I volunteer answers in my English class.	3.28	1.36
FLA4	I do not worry about making mistakes in my English class.	3.22	1.25
FLA5	I feel confident when I speak in my English class.	3.19	1.20
FLA6	I get nervous and confused when I am speaking in my English class.	3.13	1.33
FLA1	Even if I am well prepared for my English class, I feel anxious about it.	2.99	1.39
FLA2	I always feel that the other students in my class speak English better than I do.	2.88	1.44
FLA3	I can feel my heart pounding when I'm going to be asked to speak in my English class.	3.28	1.36
FLA7	I start to panic when I have to speak without preparation in my English class.	3.22	1.25

**Table 3:** Willingness to communicate.

Item ID	item	*M*	*SD*
WTC4	I am willing to make a presentation in English in front of the class.	3.06	1.28
WTC1	I am willing to voice my opinions in English to the whole classroom.	2.85	1.25
WTC3	I am willing to do a role-play in English (e.g., a job interview) standing in front of the class.	2.83	1.33
WTC7	I am willing to ask the teacher a question in English in the classroom. (Original wording: ‘I am willing to raise a question in English to the teacher in the classroom.’)	2.81	1.30
WTC2	I am willing to give a self-introduction in English to the class.	2.64	1.20
WTC9	I am willing to ask the members of my group in English how to pronounce a word in English. (Original wording: ‘I am willing to ask my group mates in English how to pronounce a word in English.’)	2.48	1.24
WTC5	I am willing to ask my teacher in English for clarification of a task that I am not sure how to do.	2.43	1.24
WTC6	I am willing to ask the teacher in English to repeat what he/she just said because I didn't understand.	2.39	1.21
WTC8	I am willing to ask the members of my group in English the meaning of a new English word that I do not know. (Original wording: ‘I am willing to ask my group mates in English the meaning of a new English word that I do not know.’)	2.18	1.14
*7_survey.docx* This file contains all survey items in both English and Ukrainian. In the survey itself, only the Ukrainian versions were used.
*8_CFA.docx* This file reports the results of a confirmatory factor analysis performed on the participants’ responses to the Likert-type items in the survey. The R code that we used for this analysis is available on request.

## 3 Experimental Design, Materials and Methods

The preliminary part of the survey provided participants with information about the project. In addition, we asked each participant to state their gender and age, and provide their email address. Email addresses were used only to verify that no participants did the survey more than once: we emphasised that we would not use this information to contact participants, or for any other purpose. All of the information in this part of the survey was translated into Ukrainian.

The main part of the survey was based on three scales; there were 22 items in all. To measure PS, we used the five items in the Students Personal Support Scale in [2]. For FLA, the Foreign Language Classroom Anxiety Scale in [3] was used. This scale consists of eight items, two of which are reverse-coded, and is an abbreviated version of the 33-item scale created by Horwitz et al. [4]. WTC was measured using the nine-item scale in [5]. Three of the items in this scale were slightly reworded for naturalness before the translations into Ukrainian were done. For each of the main survey items, the participant was asked to indicate the extent to which they disagreed or agreed with a particular statement (e.g., “I am willing to ask the teacher a question in English in the classroom”). The response options were: 1 = ‘strongly disagree’, 2 = ‘disagree’, 3 = ‘neither disagree nor agree’, 4 = ‘agree’, 5 = ‘strongly agree’. After the participant had responded to these 22 Likert-type items, they were invited to respond to the following open question (in Ukrainian in the survey, but translated into English here): “Please give specific examples of peer behaviour in class which made you feel comfortable and supported during English lessons.”

The survey was implemented online via the formr platform [7] over the period of March to May 2023, and then again from November to December 2023. To minimise any effects of item ordering (above all, a tendency to respond similarly to groups of consecutive items within the same section), all 22 Likert-type items were located on separate pages, plus the order of these pages was randomised afresh within formr for each participant. The open question always appeared last. The survey was designed in such a way that the participant was not able to skip any of the Likert-type items or the open question; consequently, there was no missing data.

A total of 483 ULEs completed the survey. The gender balance in this initial group of participants was 328 females (68 %) and 155 males (32 %). Although a bias towards female participants is by no means unusual in data sets in the area of L2 learning more generally, the female bias in the present data set was larger than usual. This may have been due, at least in part, to the fact that, at the time the data was being collected, many males in Ukraine were involved in combat in the Russo-Ukrainian War. In order to bring the gender balance into conformity with what one tends to see in L2 data sets, we randomly selected 232 females from the original pool of 328 using R. This yielded a more acceptable gender balance of 60 % female vs. 40 % male (*N* = 387; age range: 20-40; *M* = 31.2; *SD* = 7.0). These participants were adult Ukrainian nationals who were either studying English at the time in a classroom setting, or had studied it in the past in this learning context. ULEs over 40 years old were excluded from the study on the assumption that, for many individuals in this category, the learning process occurred several years earlier; hence, it might have been difficult for them to accurately recall their learning experience. The participants were recruited via Facebook: they were invited to participate via a leaflet which contained the URL for the survey. All participants completed it voluntarily.

Six outlier participants were excluded. As far as we could determine, no participant did the survey more than once. All regression assumptions were met satisfactorily.

The survey items are listed in Tables 1– 3, along with the means and standard deviations for the responses to these items. The items in each table are sorted from largest mean value to smallest.

As mentioned above, three of the items in the WTC scale were slightly reworded for naturalness. The original wordings are included in Table 3 below for comparative purposes. In WTC7, ‘raise a question in English to the teacher’ was changed to ‘ask the teacher a question in English’. In our view, the original wording was unnatural, and may have been written by a non-native speaker. In each of WTC8 and WTC9, ‘my group mates’ was changed to ‘the members of my group’. We altered this wording on the grounds that ‘mate’ can also mean ‘friend’, whereas each item was concerned instead with group membership. We do not believe that either of these rewordings altered the intended meaning of any of these three items. It is also important to bear in mind that, before it could be used, this survey needed to be translated into Ukrainian by a non-native speaker of English; therefore, the meanings of the items needed to be as clear as possible.

## Limitations

Given that the Russo-Ukrainian War was in progress while the data was being collected, we were concerned that it might not be possible for us to obtain sufficient data from this learner population during this period. For this reason, we recruited participants with a relatively wide age range.

## Ethics Statement

Prior to the survey implementation, ethics approval was obtained from The University of Southern Queensland Human Research Ethics Committee in March 2023 (HREC Project ID: ETH2023-0131). Informed consent to participate was obtained from participants. The participant indicated consent by clicking on the “Send” button at the end of the survey. All data was fully anonymised. Facebook's data redistribution policies were complied with.

## CRediT authorship contribution statement

**Gavin Austin:** Conceptualization, Methodology, Software, Validation, Writing – review & editing, Visualization, Formal analysis, Resources, Data curation, Supervision. **Andriy Yanovych:** Conceptualization, Investigation, Writing – original draft, Project administration.

## Data Availability

Dataset for a project on affect and conation in second language learning with Ukrainian learners of English (Original data) (OSF)
